# Understanding public support for COVID-19 pandemic mitigation measures over time: Does it wear out?

**DOI:** 10.3389/fpubh.2023.1079992

**Published:** 2023-03-03

**Authors:** John B. F. De Wit, Denise T. D. de Ridder, Wijnand van den Boom, Floor M. Kroese, Bas van den Putte, F. Marijn Stok, Mariken Leurs, Marijn de Bruin

**Affiliations:** ^1^Department of Interdisciplinary Social Science, Utrecht University, Utrecht, Netherlands; ^2^Department of Social, Health and Organizational Psychology, Utrecht University, Utrecht, Netherlands; ^3^Corona Behavioural Unit, National Institute for Public Health and the Environment (RIVM), Bilthoven, Netherlands; ^4^Amsterdam School of Communication Research, University of Amsterdam, Amsterdam, Netherlands; ^5^Radboud Institute for Health Sciences, Radboud University Medical Center, Nijmegen, Netherlands

**Keywords:** COVID-19, mitigation measures, health protection, health behavior (MeSH), public support, pandemic fatigue

## Abstract

**Background:**

COVID-19 mitigation measures intend to protect public health, but their adverse psychological, social, and economic effects weaken public support. Less favorable trade-offs may especially weaken support for more restrictive measures. Support for mitigation measures may also differ between population subgroups who experience different benefits and costs, and decrease over time, a phenomenon termed “pandemic fatigue.”

**Methods:**

We examined self-reported support for COVID-19 mitigation measures in the Netherlands over 12 consecutives waves of data collection between April 2020 and May 2021 in an open population cohort study. Participants were recruited through community panels of the 25 regional public health services, and through links to the online surveys advertised on social media. The 54,010 unique participants in the cohort study on average participated in 4 waves of data collection. Most participants were female (65%), middle-aged [57% (40–69 years)], highly educated (57%), not living alone (84%), residing in an urban area (60%), and born in the Netherlands (95%).

**Results:**

COVID-19 mitigation measures implemented in the Netherlands remained generally well-supported over time [all scores >3 on 5-point scale ranging 1 (low)−5 (high)]. During the whole period studied, support was highest for personal hygiene measures, quarantine and wearing face masks, high but somewhat lower for not shaking hands, testing and self-isolation, and restricting social contacts, and lowest for limiting visitors at home, and not traveling abroad. Women and higher educated people were more supportive of some mitigation measures than men and lower educated people. Older people were more supportive of more restrictive measures than younger people, and support for more socially restrictive measures decreased most over time in higher educated people or in younger people.

**Conclusions:**

This study found no support for pandemic fatigue in terms of a gradual decline in support for all mitigation measures in the first year of the pandemic. Rather, findings suggest that support for mitigation measures reflects a balancing of benefits and cost, which may change over time, and differ between measures and population subgroups.

## Background

Since late 2019, when the Coronavirus Disease 2019 (COVID-19) virus was first reported, it has traveled rapidly within and across countries, causing serious illness in more vulnerable infected people and resulting in millions of deaths worldwide (1). To mitigate the enormous population health impact of the COVID-19 pandemic, governments globally were quick to encourage or mandate a range of unprecedented mitigation measures (2), including personal hygiene behaviors (e.g., handwashing, covering coughs and sneezes), wearing masks covering mouth and nose, and limiting social interactions (e.g., physical distancing, staying at home, quarantining/self-isolating). While the aim of these mitigation measures is to protect population health, they can also adversely affect people's psychological, social, and economic outcomes. People differ in their preferences for the potential trade-offs between public health and other outcomes of COVID-19 mitigation measures and that may have implications for policy decisions (3). Notably, resistance to mitigation measures can undermine pandemic control (4). The public's acceptance of COVID-19 mitigation measures [i.e., the extent to which people consider measures justified (5)], is critical to their implementation and effect (6). The overall aim of the present study is to contribute to a better understanding of public support for COVID-19 pandemic mitigation measures, that is, to what extent the public hold favorable views of measures [e.g., find these acceptable (5, 6) or important (4), or support (vs. oppose) (7) or agree (vs. disagree) with them (8)]. More specifically, this study aimed to assess the dynamics of public support, including which measures are more likely to be supported, for how long and by whom, providing new knowledge that can guide policy decisions for effective pandemic control.

Cross-sectional self-report surveys of representative population samples in New Your City (*N* = 286), Los Angeles (*N* = 259) and across the United States (US; *N* = 1,676), undertaken between 5 and 12 May 2020, found most people supported stay-at-home-orders (79.5%), non-essential business closures (67.3%), keeping physical distance (87.7%), limiting group gatherings to 10 people (82.4%), and not allowing dining inside restaurants (66.6%) (9). Self-report surveys in a nationally representative cohort of US adults conducted in April (*N* = 1,468), July (*N* = 1,337), and November 2020 (*N* = 1,222) found that while support for social distancing had dropped from 89% in April to 79% in July, this remained stable in November 2020 at 78%. Also, in July and November, more than three quarters of respondents supported mask wearing and nearly as many supported contact tracing (4). Outside the US, a cross-sectional self-report survey undertaken in November 2020 in a representative sample of the Hungarian population (*N* = 1,000), found high support for preventive measures, if implemented through regulations as well as nudges, to promote hand hygiene, social distancing and face mask wearing (mean scores range 5.2–5.9, assessed on a 7-point scale from 1 = certainly oppose to 7 = certainly support) (7). A first self-report survey in a consumer panel (*N* = 1,654) conducted in the German-speaking part of Switzerland between late March and early April 2020, found overall high support for mitigation measures (mean score = 5.74, assessed on a 7-point scale from 1 = do not agree at all to 5 = completely agree), as averaged across four measures (i.e., closure of schools, closure of restaurants and bars, discouraging people from leaving the house, closure of all shops except grocery shops and pharmacies) (5). A second self-report survey in this panel (*N* = 1,267), conducted in the second half of April 2020, found a decrease in support for these implemented measures (6).

A two-phase self-report survey in a population panel (phase 1, June 2020 *N* = 212, phase 2, December 2020, *N* = 150) in the United Kingdom (UK) (8), found that support varied substantially according to the specific COVID-19 mitigation measures. Support for some measures was initially high and, although declining somewhat, remained favorable (mean scores > 3– <5, assessed on an 11-point scale from −5 = strongly oppose to +5 = strongly support) (i.e., handwashing after every outside trip, banning all public gatherings, banning visiting anyone outside your household, closing all pubs and restaurants, closing all non-essential shops). However, for other measures (i.e., restricting exercise outside to once per day, restricting visitor access to hospitals for terminally ill patients, restricting visitor access to hospitals for maternity wards, restricting attendance at funerals) lower initial support was found (mean scores <3 to >1), which further decreased at phase 2 (mean scores <1 to >-0.5). These differences in support for COVID-19 mitigation measures may reflect that some measures are easier to implement than others (8). Also, some measures may have a limited impact on people's daily lives (e.g., frequent hand washing), while others (e.g., limiting social contacts, working from home) can constrain people's usual behavior and limit their choices (10).

Surveys into the extent of support for COVID-19 mitigations measures have also assessed and found evidence for a diversity of factors related to differences in support. A cross-sectional survey in a representative sample in Hungary found that people with a higher level of risk perception (i.e., composite measure encompassing level of worry, perceived likelihood of direct effects for self or family members and friends, beliefs about how many people in the country will be affected, and perceived probability of falling ill and falling ill seriously) were more supportive of mitigation policies (7). Also, people who themselves had contracted COVID-19 or had a close friend or family member who had contracted COVID-19 reported a higher level of risk perception (7). A two-wave self-report survey in a population panel in the UK found that people's perceived health threat to them personally or to close others (e.g., “I think it is likely a close family member will die from COVID-19 at some point in the future”) did not influence their current or future support for policies, while support was related to perceived general health threat (e.g., “I think the number of deaths directly caused by COVID-19 is a massive threat to this country”) (8). Assessing a broader range of potential factors of influence, multivariable regression analyses of data obtained in the three-wave panel study conducted between April and November 2020 in New York City, Los Angeles and the US more broadly found that support for social distancing, mask-wearing, and contact tracing was lower in young adults (18–34 years) compared to older age groups, people with a republican or independent political affiliation compared to a democratic political affiliation, and people with a lot of trust in science compared to those with some or not a lot of trust (4). In addition, support for contact tracing was lower in people with a more fixed (i.e., authoritarian, obedient) worldview compared to a more fluid (i.e., non-authoritarian, self-reliant) worldview (i.e., beliefs about how to relate to others) (4).

Using multivariable regression analysis, a longitudinal assessment of a range of factors potentially related to support across four mitigation measures in a two-wave survey in Switzerland found that support at wave 2, controlling for support at wave 1, was higher among people with higher general confidence (i.e., a psychological buffer for coping with uncertainty), people perceiving more health risk from COVID-19 (i.e., composite measure encompassing perceived risk of infection for self and family members or acquaintances, concern about fatalities in one's social environment and the country, and concern about overload of the healthcare system) and people reporting higher social trust (i.e., value similarity) in the Swiss government and the pharmaceutical industry (6). Support for measures was lower in people who reported higher general trust (i.e., the belief that most people are trustworthy most of the time), in people who scored higher on a measure of individualism as an indicator of cultural worldview, and in people who more strongly believed that tradeoffs between expected benefits and potential economic, educational, and social costs of mitigation measures were not sufficiently considered (6). Factors that influence support for mitigation measures, including social and political trust, perceived risk and perceived trade-offs can differ between population groups and contribute to differences in support for measures between population groups. Differences in the balance of benefits and costs of COVID-19 mitigation measures in particular may explain differences in support between age groups. Adolescents and young adults are considered particularly likely to be less supportive of COVID-19 measures, as their personal risk of serious health impacts is low while the adverse impact they experience may be high (11–13).

Support for COVID-19 mitigation measures may also be affected by the duration of restrictions, and potentially decrease over time. Experimental research in the general population in Germany showed that public acceptance of a lockdown was mostly reduced by increasing duration and was not much affected by the extensiveness or flexibility of restrictions (14). Also, a population survey of views on potential lockdown scenarios in Spain found that willingness to be confined decreased as duration increased (15). Monitoring support for implemented COVID-19 mitigation measures, population surveys in the US showed decreasing support for social distancing between April and June 2020, with support remaining stable by November 2020 (4). Support for in-door face mask wearing and contact tracing was only assessed in June and November 2020 and found to be stable at a level similar to the already decreased level of support for social distancing at those times. A two-wave population survey in the UK found that although people on average continued to support all assessed measures, the strength of support decreased in the first year of the pandemic (8). Monitoring adherence to mandated mitigation measures, population surveys in five cities in Australia, the UK, and the US found decreasing adherence to mask wearing and other mitigation measures (16). A pooled analysis of publicly available data from 14 countries (Canada, Denmark, Finland, France, Germany, Italy, Japan, Netherlands, Norway, Singapore, South Korea, Spain, Sweden, and UK), in contrast, found increasing adherence to face mask wearing, which authors consider “low cost and arguably habituating” [(17), p. 1146]. This 14-country analysis also found declined adherence to avoidance of gatherings and avoidance of going out, considered “behaviors that entail high and potentially cumulative individual costs over time” [(17), p. 1146]. However, this decline slowed over time and support partially rebounded (17). Analysis of mobile phone data from 124 countries also showed a decrease and then rebound in adherence to mobility restrictions, indicated by time spent in residential locations, and on retail and recreation visits (17).

Potentially decreasing support for (15) and adherence to (16, 17) COVID-19 mitigation measures has been suggested to reflect pandemic fatigue. According to the World Health Organization (WHO), pandemic fatigue is a “demotivation to follow recommended protective behaviors, emerging gradually over time and affected by a number of emotions, experiences and perceptions” [(18), p. 7], comprising decreased support for and adherence to COVID-19 mitigation measures (18). Pandemic fatigue is noted to have gained prominence as an explanation of a decline in following the rules to prevent the spread of COVID-19 (19). However, its occurrence is contested (19–21). We found two studies that explicitly aimed to assessed evidence of pandemic fatigue based on trends in adherence to COVID-19 mitigation measures, of which one did not find the continual gradual decrease in adherence (17). The other compared adherence in only two periods (16), which may have compounded reporting bias. Three further population surveys assessed trends in support for COVID-19 mitigation measures and provide mixed evidence for pandemic fatigue, albeit that this was not explicitly addressed. More specifically, two-wave surveys in the UK and Switzerland found high but somewhat declining support in the first year of the pandemic (5, 8), while a three-wave assessment in the US found declining and then stabilizing support (4). The importance of longer-term follow-up over multiple assessments to assess pandemic fatigue is underscored by a multi-country study (17), which noted a rebound in support for social distancing measures over a longer period. This may reflect the noted importance of the evolving health threat of COVID-19 in understanding changing patterns of support (20), echoed by evidence that the perceived health risk (5) or general threat (8) of COVID-19 is associated with continued support for mitigation measures.

Despite the importance of understanding trends and differences in the public's support for COVID-19 mitigation measures for the successful easing of the pandemic, to date there is little population-based research that directly examines longer-term trends in support for COVID-19 mitigation measures, and there is a particular lack of research reporting on data collected in a prospective cohort study. There is also a dearth of research comparing trends in support according to different COVID-19 mitigation measures and assessing differences in evolving support between population subgroups. To address these critical knowledge gaps, we draw on data from a population cohort study in The Netherlands. We examined support for non-vaccination COVID-19 mitigation measures that were implemented in The Netherlands between April 2020, when COVID-19 was declared a pandemic by the World Health Organization (18), and May 2021, when the roll-out of the COVID-19 vaccination program was well underway in The Netherlands. We specifically assessed differences in trends in support according to type of mitigation measure and expect that if pandemic fatigue occurs, this will be evident in gradual decreases in support for all measures, with stronger decreases for measures with more impact on people's daily lives, in particular substantial restrictions in social contacts (3–5, 14). We also expect that the extent and trends in support for measures differs between population subgroups, reflecting the balance of expected health benefits due to personal health risks of COVID-19 and cost due to the high impact of mitigation measures on people's daily life. Support may especially be lower and decline more rapidly among young people (4), whose social lives can be substantially curtailed by mitigation measures (13), while their personal health risk is generally low (11, 12).

## Methods

### Data and sample

The study methods are summarized here and described in more detail elsewhere (22). This study used data collected at 12 points in time between April 2020 and May 2021 in an open cohort to assess support for COVID-19 mitigation measures in The Netherlands. The cohort study is jointly undertaken by the National Institute for Public Health and the Environment (RIVM) and the Netherlands Association of Regional Public Health Services and Regional Medical Emergency Preparedness and Planning Offices (GGD GHOR). Data was collected through self-completion online questionnaires, fielded by a research agency (Research 2Evolve). The timing of waves and the numbers of participants per wave are shown in Supplementary Table 1. All participants provided online informed consent before filling out each questionnaire.

In April 2020, individuals aged 16 years and older who participated in an existing community health and wellbeing panel of one of the 25 regional public health services in The Netherlands, or in a similar panel of one of eight municipalities, were invited by email to participate in a survey of behavior and subjective wellbeing during the corona pandemic. Each of the existing panels consisted of 1,000–10,000 participants who completed online surveys on health-related topics or other topics several times per year. Participants in these existing community panels were mostly recruited from consenting participants in representative population samples completing statutory public health surveys jointly undertaken by the Netherlands Association of Regional Public Health Services and Regional Medical Emergency Preparedness and Planning Offices, the National Institute for Public Health and the Environment and Statistics Netherlands (CBS, the government agency for statistics). Additional panel participants were recruited from consenting participants in other regional or local surveys and activities, or through self-referral.

Participants who completed the first survey of the cohort study were asked if they consented to receiving invitations for future survey rounds. To compensate for dropout over time, additional participants were recruited in about half of the rounds of data collection, with timing of further recruitment depending on holiday periods, staff availability and other practical issues (e.g., timing of the 2022 periodic statutory public health survey). The focus of additional recruitment was on people from population subgroups underrepresented in the cohort, particularly individuals aged 16–24 years (22). Additional participants were recruited through social media (e.g., Facebook) and mailing lists of non-government organizations, including those that serve young people, such as the National Youth Council (NJI) and the Council for Secondary Vocational Education and Training (MBO Raad).

### Measures

At baseline, participants completed an extensive assessment of demographic characteristics (e.g., age, educational level, gender, country of birth, living and work situation), socioeconomic status (e.g., unemployment, change in financial situation due to the COVID-19 pandemic), health status (e.g., having a pre-existing physical medical health condition), and COVID-19 vaccination, testing and infection. To reduce participation burden, participants were then randomly assigned to a subset of modules containing questions on specific themes; module assignment remained consistent in follow-up surveys. In follow-up surveys, participants completed brief assessments of COVID-19 vaccination, testing and infection, and of sociodemographic characteristics that could have changed or were required to enable linkage of individual's data across surveys. Participants were eligible for the current study if they completed the module containing questions on support for COVID-19 mitigation measures as implemented at the time of the specific wave of data collection.

#### Demographic characteristics

Surveys included questions about participants sex (male/female), age (16–24, 25–39, 40–55, 55–69, and 70+), educational level (low, middle, high, according to standards in The Netherlands), living alone (yes/no), country of birth (born in the Netherlands or not), and whether people had a medical condition that put them at increased risk of severe health impact of COVID-19 (medical condition yes/no).

#### Outcome measures

We assessed support for 16 COVID-19 mitigation measures mandated by the government of The Netherlands in the period April 2020–May 2021 (see Table 1). Support for specific measures was only assessed in a wave when they were implemented at the time of data collection. The phrasing of items assessing support for a measure was adjusted when a measure changed (e.g., the numbers of visitors permitted at home varied over time). For each measure, participants were asked to indicate their extent of support on a scale from 1 (not at all) to 5 (completely); they could also choose “no opinion” (recoded as missing for current analyses).

**Table 1:** Item loadings^*^ and internal consistency of five multi-item components^#^ resulting from principal component analysis.

**Support for COVID-19 mitigation measure**	**Personal hygiene**	**Testing and self-isolation**	**Quarantine**	**Restricting social contacts**	**Wearing face masks**
Use paper tissue for cleaning nose	0.813				
Cover cough or sneeze by elbow	0.793				
Wash hands for 20 s with water and soap	0.712				
Stay at home when experiencing symptoms		0.814			
Get tested for COVID-19 when having symptoms		0.790			
Stay at home when symptoms in household contact		0.560			
Quarantine after visiting high-risk country or area			0.818		
Quarantine after close contact with infected person			0.738		
Work from home as much as possible				0.844	
Avoid crowded places				0.702	
Keep 1.5 m distance from others				0.641	
Wearing face mask in public spaces					0.893
Wearing face mask in public transport					0.870
Internal consistency	Cronbach's alpha = 0.74	Cronbach's alpha = 0.80	Cronbach's alpha = 0.84	Cronbach's alpha = 0.82	Cronbach's alpha = 0.95

* Item loadings are shown after Varimax rotation with Kaiser Normalization; item loadings below 0.50 are suppressed.

# Items not included in a multi-item component: not shaking hands, limiting visitors at home, and not traveling abroad.

### Data analysis

Statistical analyses were performed using Stata (version 16.1; Stata Corp., College Station, Texas, USA) and R (version 4.2). To reduce the number of dependent variables, a principal components analysis (PCA) was performed on 14 COVID-19 mitigation measures support items. Two items, Not Shaking Hands and Limiting Visitors at Home, were not included in the PCA because of their relevance as stand-alone items. Based on the criterion of eigenvalue greater than one, five multi-item components were identified: Personal Hygiene, Testing and Self-Isolation, Quarantine, Restricting Social Contacts and Wearing Face Masks (see Table 1), as well as one single items: not traveling abroad. To determine the loadings on the five multi-item components, a confirmatory PCA with orthogonal (Varimax) rotation was performed, as shown in Table 1.

Multivariable linear mixed model analyses were performed to assess the association between each of the eight outcome measures and gender, age, educational level, living arrangement, country of birth, and medical condition. Due to variability over time in infection rates and strictness of COVID-19 prevention measures, we allowed for non-linearity of the associations over time by including time (wave of data collection) as a categorical variable. All models also included a random intercept for study participants and a random slope for time, as well as an unstructured covariance matrix. Because of multiple testing and the large sample size, associations were considered relevant when the *p*-value was <0.001, and the magnitude b-coefficient was *b* ≥ |0.20| (reflecting a 5% points or larger change in the outcome variable). To assess differences between population subgroups in trends in support over time, interaction terms between time and gender, age, educational level, living arrangement, country of birth, and medical condition were included. To limit the number of interaction terms in the models, differences in trends were assessed separately for each subgroup characteristic. Missing data were handled using complete case analysis. We assume that data are missing at random (MAR), with missingness related to assessed participant characteristics. To account for participant drop-out, we included participant characteristics related to (dis)continued participation into the analyses.

## Results

### Participants' demographic characteristics

The 54,010 unique participants in the cohort study on average participated in 4 waves of data collection (SD = 4, Median = 2, IQR = 1.7). Most participants were female (65%), middle-aged [57% (40–69 years)], highly educated (57%), not living alone (84%), residing in an urban area (60%), and born in the Netherlands (95%). A minority of 24% reported to have an underlying medical condition. Participants' demographic characteristics are shown in Table 2.

**Table 2:** Participants' demographic characteristics (*N* = 54,010^*^).

	***N*** **(%)**
**Sex**	
Female	35,055 (65%)
Male	18,862 (35%)
**Age**	
16–24 years	2,783 (5%)
25–39 years	12,603 (23%)
40–54 years	15,540 (29%)
55–69 years	15,273 (28%)
70+ years	7,811 (14%)
**Educational level**	
Low	6,815 (13%)
Middle	16,060 (30%)
High	30,517 (57%)
**Living alone**	
No	45,333 (84%)
Yes	8,677 (16%)
**Country of birth**	
The Netherlands	51,222 (95%)
Other	2,675 (5%)
**Medical condition**	
No	40,798 (76%)
Yes	13,107 (24%)

* Due to missing values, for some variables numbers may not add up to the overall total number of unique participants.

### Support for COVID-19 mitigation measures

**Figure 1:**
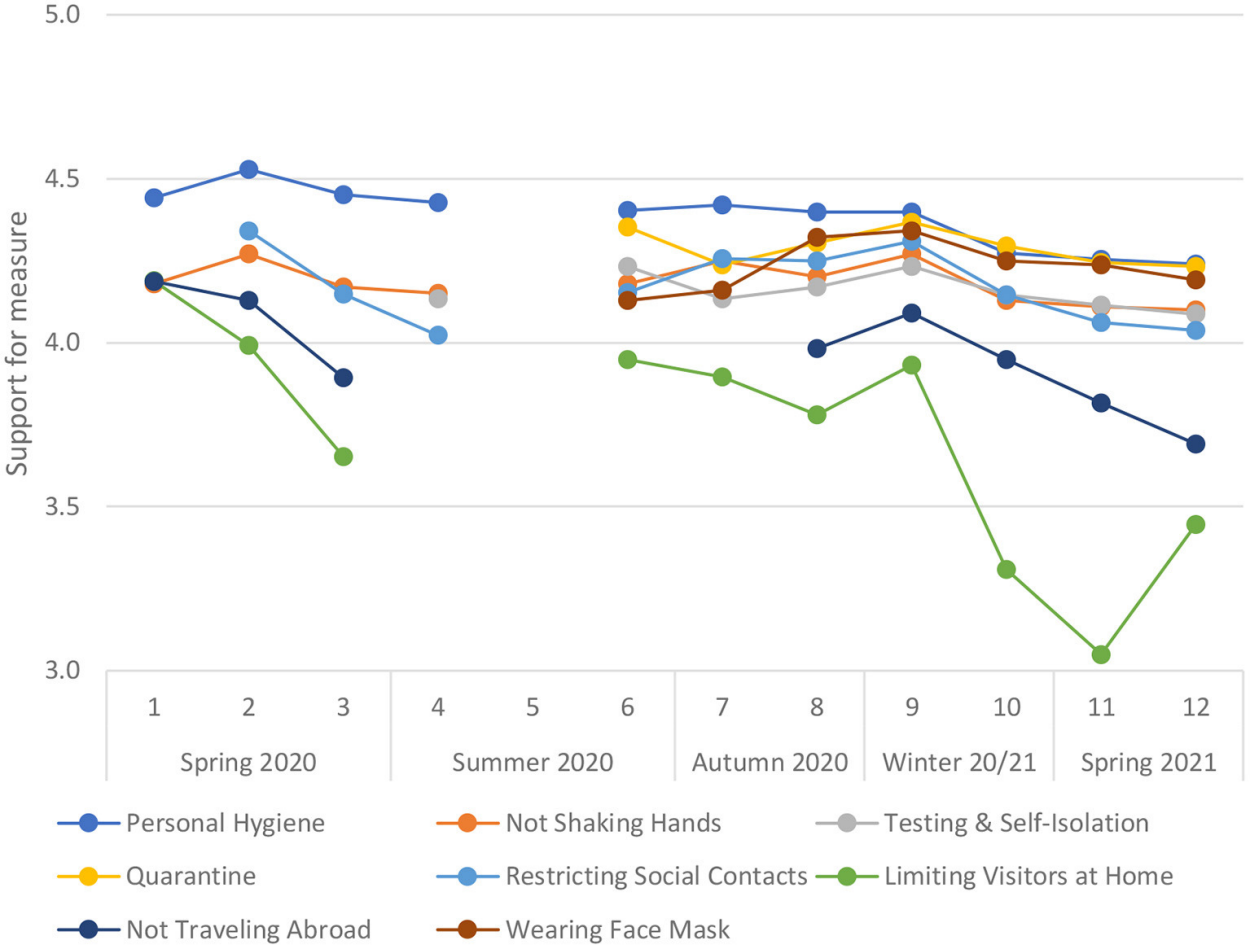
Support for COVID-19 mitigation measures over time. Wave 1: April 2020. Wave 12: May 2021; *N* = 54,010 participants who responded to support items; analyses of trends corrected for participant demographic characteristics.

Figure 1 shows that, on average, COVID-19 mitigation measures implemented in the Netherlands were generally well-supported across the waves in which support was assessed. Overall, support was highest for personal hygiene, quarantine and wearing faces masks, high but slightly lower for not shaking hands, testing and self-isolation, and restricting social contacts, and lowest for limiting visitors at home, and not traveling abroad.

Table 3 shows that, based on the criterion of *b* ≥ |0.20|, support for personal hygiene measures was stable but decreased somewhat toward the end of the study period. Support for not shaking hands, testing and self-isolation, and quarantine remained stable. Support for restricting social contacts was somewhat variable and overall decreased over the study period. A substantially variable and overall decreasing pattern in support was observed for the limitation of the number of visitors allowed at home, which repeatedly changed over time and ranged between 6 to 1 person per day. Support for restrictions on traveling abroad varied over time and declined overall. Support for wearing face masks initially increased and remained stable thereafter.

**Table 3:** Multivariable analysis of trends in support of COVID-19 mitigation measures over time.

		**Personal hygiene**	**Not shaking hands**	**Testing and self-isolation**	**Quarantine**	**Restricting social contacts**	**Limiting visitors at home**	**Not traveling abroad**	**Wearing face mask**
		**b (95% CI)**	**b (95% CI)**	**b (95% CI)**	**b (95% CI)**	**b (95% CI)**	**b (95% CI)**	**b (95% CI)**	**b (95% CI)**
Spring 2020	Wave 1	Reference	Reference				Reference	Reference	
	Wave 2	0.08 (0.08 to 0.09)	0.09 (0.08 to 0.10)			Reference	−0.20 (−0.21 to −0.18)	−0.06 (−0.07 to −0.04)	
	Wave 3	0.01 (0.001 to 0.01)	−0.004 (−0.02 to 0.01)			−0.19 (−0.20 to −0.18)	−0.54 (−0.55 to −0.52)	−0.29 (−0.31 to −0.28)	
Summer 2020	Wave 4	−0.02 (−0.02 to −0.01)	−0.02 (−0.04 to −0.01)	Reference		−0.33 (−0.34 to −0.32)			
	Wave 5								
	Wave 6	−0.04 (−0.05 to −0.03)	0.001 (−0.01 to 0.01)	0.08 (0.07 to 0.09)	Reference	−0.20 (−0.21 to −0.19)	−0.24 (−0.25 to −0.22)		
Autumn 2020	Wave 7	−0.02 (−0.03 to −0.01)	0.07 (0.06 to 0.09)	−0.01 (−0.02 to −0.001)	−0.12 (−0.13 to −0.11)	−0.09 (−0.10 to −0.08)	−0.29 (−0.31 to −0.28)		Reference
	Wave 8	−0.04 (−0.05 to −0.04)	0.02 (0.005 to 0.03)	0.02 (0.01 to 0.03)	−0.05 (−0.06 to −0.04)	−0.10 (−0.11 to −0.09)	−0.40 (−0.42 to −0.39)	−0.20 (−0.22 to −0.19)	0.27 (0.26 to 0.29)
Winter 20/21	Wave 9	−0.05 (−0.05 to −0.04)	0.09 (0.08 to 0.11)	0.09 (0.07 to 0.10)	0.01 (0.002 to 0.02)	−0.04 (−0.05 to −0.03)	−0.25 (−0.27 to −0.24)	−0.10 (−0.11 to −0.08)	0.29 (0.27 to 0.30)
	Wave 10	−0.17 (−0.18 to −0.16)	−0.05 (−0.06 to −0.03)	−0.004 (−0.02 to 0.01)	−0.06 (−0.07 to −0.05)	−0.20 (−0.21 to −0.18)	−0.88 (−0.90 to −0.86)	−0.24 (−0.26 to −0.22)	0.20 (0.18 to 0.21)
Spring 2021	Wave 11	−0.19 (−0.20 to −0.18)	−0.07 (−0.09 to −0.05)	−0.04 (−0.05 to −0.03)	−0.11 (−0.12 to −0.10)	−0.28 (−0.30 to −0.27)	−1.14 (−1.16 to −1.12)	−0.37 (−0.39 to −0.35)	0.18 (0.17 to 0.20)
	Wave 12	−0.20 (−0.21 to −0.19)	−0.08 (−0.10 to −0.06)	−0.07 (−0.08 to −0.05)	−0.12 (−0.13 to −0.11)	−0.31 (−0.33 to −0.30)	−0.74 (−0.76 to −0.72)	−0.49 (−0.51 to −0.48)	0.15 (0.13 to 0.16)
Number of observations	200,023	201,132	130,677	114,778	159,371	184,781	149,862	88,517	

### Differences between population subgroups

We also examined whether support for COVID-19 mitigation measures differed between population subgroups. Table 4 shows that women on average reported more support than men for measures regarding personal hygiene, not shaking hands, quarantine, and not traveling abroad. Compared to people with a lower level of education, people with a higher level of education expressed more support for measures with respect to personal hygiene, not shaking hands, testing and self-isolation, restricting social contacts, and not traveling abroad. The largest differences were observed for age. The younger groups (16–24 and 25–39 years) reported substantially lower support than older groups (in particular the 70+ years reference group) for testing & self-isolation, quarantine, restricting social contact, limiting visitors at home, and wearing face masks. Support for personal hygiene measures and not shaking hands was, however, higher in the youngest age group. We found no differences in support for COVID-19 mitigation measures according to country of birth, living arrangements, or underlying medical condition.

**Table 4:** Multivariable analysis of differences in support of COVID-19 mitigation measures according to participants' demographic characteristics.

	**Personal hygiene**	**Not shaking hands**	**Testing and self-isolation**	**Quarantine**	**Restricting social contact**	**Limiting visitors at home**	**Not traveling abroad**	**Wearing face mask**
	**b (95% CI)**	**b (95% CI)**	**b (95% CI)**	**b (95% CI)**	**b (95% CI)**	**b (95% CI)**	**b (95% CI)**	**b (95% CI)**
**Sex**								
Female	Reference	Reference	Reference	Reference	Reference	Reference	Reference	Reference
Male	−0.30 (−0.31 to −0.29)	−0.25 (−0.26 to −0.23)	−0.13 (−0.14 to −0.11)	−0.23 (−0.25 to −0.21)	−0.19 (−0.21 to −0.18)	−0.15 (−0.17 to −0.13)	−0.25 (−0.27 to −0.23)	−0.17 (−0.19 to −0.14)
**Age**								
70+	Reference	Reference	Reference	Reference	Reference	Reference	Reference	Reference
55–69 years	0.14 (0.12 to 0.15)	0.22 (0.19 to 0.24)	−0.03 (−0.06 to −0.01)	−0.08 (−0.10 to −0.05)	−0.15 (−0.17 to −0.12)	−0.13 (−0.16 to −0.10)	0.08 (0.05 to 0.11)	−0.17 (−0.21 to −0.14)
40–54 years	0.16 (0.14 to 0.17)	0.17 (0.14 to 0.20)	−0.19 (−0.21 to −0.16)	−0.27 (−0.30 to −0.24)	−0.38 (−0.41 to −0.36)	−0.38 (−0.41 to −0.35)	−0.06 (−0.09 to −0.03)	−0.51 (−0.54 to −0.47)
25–39 years	0.17 (0.16 to 0.19)	0.13 (0.11 to 0.16)	−0.29 (−0.31 to −0.26)	−0.34 (−0.36 to −0.31)	−0.48 (−0.50 to −0.45)	−0.67 (−0.70 to −0.64)	−0.14 (−0.17 to −0.10)	−0.74 (−0.78 to −0.70)
16–24 years	0.25 (0.22 to 0.28)	0.24 (0.19 to 0.28)	−0.25 (−0.29 to −0.21)	−0.34 (−0.38 to −0.30)	−0.51 (−0.55 to −0.47)	−0.84 (−0.89 to −0.79)	−0.13 (−0.18 to −0.08)	−0.65 (−0.71 to −0.59)
**Educational level**								
Low	Reference	Reference	Reference	Reference	Reference	Reference	Reference	Reference
Middle	0.09 (0.08 to 0.11)	0.35 (0.32 to 0.38)	0.08 (0.05 to 0.10)	0.03 (0.001 to 0.06)	0.11 (0.08 to 0.14)	0.04 (0.01 to 0.07)	0.27 (0.24 to 0.30)	0.03 (−0.01 to 0.07)
High	0.21 (0.19 to 0.22)	0.68 (0.65 to 0.70)	0.22 (0.19 to 0.24)	0.10 (0.08 to 0.13)	0.34 (0.31 to 0.36)	0.17 (0.14 to 0.20)	0.42 (0.39 to 0.45)	0.16 (0.13 to 0.20)
**Country of birth**								
The Netherlands	Reference	Reference	Reference	Reference	Reference	Reference	Reference	Reference
Other	0.04 (0.02 to 0.07)	0.02 (−0.02 to 0.05)	0.08 (0.04 to 0.11)	0.003 (−0.03 to 0.04)	0.12 (0.08 to 0.15)	0.18 (0.14 to 0.22)	−0.15 (−0.19 to −0.10)	0.18 (0.13 to 0.23)
**Living alone**								
No	Reference	Reference	Reference	Reference	Reference	Reference	Reference	Reference
Yes	−0.03 (−0.04 to −0.01)	−0.05 (−0.07 to −0.03)	−0.03 (−0.05 to −0.02)	−0.03 (−0.05 to −0.01)	−0.04 (−0.06 to −0.03)	0.005 (−0.02 to 0.03)	−0.03 (−0.05 to −0.01)	−0.05 (−0.08 to −0.03)
**Medical condition**								
No	Reference	Reference	Reference	Reference	Reference	Reference	Reference	Reference
Yes	0.01 (−0.003 to 0.01)	−0.01 (−0.02 to 0.004)	0.02 (0.01 to 0.03)	0.05 (0.03 to 0.06)	0.04 (0.03 to 0.05)	0.05 (0.03 to 0.07)	0.03 (0.01 to 0.04)	0.04 (0.02 to 0.06)
Number of observations	200,023	201,132	130,677	114,778	159,371	184,781	149,862	88,517

We found several differences in trends in support for measures between population subgroups (see Supplementary Table 2). Compared to people with a low level of education, support for not shaking hands and not traveling abroad decreased more strongly among people with a high level of education. Also, compared to the oldest age group, support in younger age groups for measures related to not shaking hands, restricting social contacts, and limiting visitors at home decreased more strongly over time.

## Discussion

The aim of this study was to assess the extent and trends in public support for COVID-19 mitigation measures in The Netherlands, including according to type of measure and in population subgroups. We found substantial public support for implemented measures during the whole period monitored, despite variability over time in infection rates and strictness of COVID-19 prevention measures. However, whereas for several measures support was stable, support for others varied during the pandemic. Also, support for several measured showed a declining trend over time. We found that when support for measures declined, this was strongest for higher educated people and for younger people. Taken together, our findings do not provide evidence for general pandemic fatigue over time. Rather, they suggest that reductions in support for COVID-19 mitigation measures reflect differences in trade-offs between specific measures and for different population subgroups. The potentially adverse impacts of specific measures may outweigh the experienced benefits for some population subgroups, especially younger age groups.

To date, few studies have reported data on trends in support for COVID-19 mitigation measures and these concluded that, despite some decreases, support overall remained strong in the first year of the pandemic in the US (4), Switzerland (6), and the UK (8). Some other studies have also reported on differences in support for COVID-19 mitigation measures between population subgroups and differences between population groups in trends in support. Findings of previous research are mixed with respect to gender differences in support for COVID-19 mitigation measures. A cross-sectional population survey in the US found that women were more supportive than men of some measures (9), while a longitudinal three-wave panel survey in the US generally found no significant differences, with one exception being an observed higher level of support among women for contact tracing in the November 2020 wave (4). A two-wave survey in Switzerland also did not find any gender differences in support for COVID-19 mitigation measures and gender (5, 6). Furthermore, whereas we found some differences in support according to participants' educational level, no significant associations between educational level and support for measures were found in a longitudinal three-wave panel survey in the US (4).

We observed most and largest differences in support for COVID-19 mitigation measures between participants of different age groups. The younger age groups reported substantially lower support than the older age groups for testing and self-isolation, quarantine, restricting social contact, limiting visitors at home, and wearing face masks. A longitudinal three-wave panel survey in the US also found that, compared to younger age groups, support for COVID-19 mitigation measures was higher in older and oldest age groups (4), while a two-wave panel survey in Switzerland found no significant association between age and support for mitigation measures (5, 6). A cross-sectional survey in the US reported mixed findings regarding age-related differences in support for specific measures (9). Of note, we found that support for personal hygiene measures and not shaking hands was higher in the youngest age group. This suggest the possibility that, contrary to previous suggestions (13), support for and compliance with COVID-19 mitigation measures is not inevitably lower among young people.

Our findings contribute novel evidence to the ongoing debate on potential pandemic fatigue. Pandemic fatigue has been explicitly assessed in studies of trends in adherence to, rather than support of, mitigation measures, which found methodologically limited (16) or no evidence (17) for potential pandemic fatigue. Furthermore, trends in support for COVID-19 measures to date was assessed in surveys that encompassed two or three waves of data collection over a maximum time span of seven months and do not provide evidence on longer term trends over time. Our study assessed trends in support for COVID-19 mitigation measures at a larger number of time points over a period of one year and did not find the gradual decline in support for all measures that would indicate pandemic fatigue. Nevertheless, we did find decreasing support for three of the eight COVID-19 mitigation measures we assessed: personal hygiene measures, not traveling abroad, and limitation of the number of visitors allowed at home. Furthermore, we found that support for not shaking hands and not traveling abroad decreased more strongly among people with a high level of education than in people with a low level of education. Also, support in younger age groups for not shaking hands, restricting social contacts, and limiting visitors at home decreased more strongly than in the oldest age group.

The differing trends in support we observed for specific mitigation measures in specific population subgroups are aligned with differences and changes in the perceived trade-offs in the balance of potential benefits and costs of specific measures over time. The importance of differences in perceived trade-offs in understanding differences in support for mitigation measures is underscored by experimental research (3, 14), as well as population surveys of population support showing that support for COVID-19 mitigation is lower and decreases when people more strongly believe that economic, educational, and social costs are insufficiently weighed (5, 6). Further evidence for the importance of potential benefits and costs in understanding differences in support for mitigation measures is provided by research that found that more support for and adherence to COVID-19 mitigation measures is associated with a higher perceived need for such measures (20), as indicated by concern about COVID-19 (11), experienced public health danger or personal health threat of COVID-19 (8, 10, 12), or perceived health risks of COVID-19 to self or important others (5–7). In addition, research reporting associations between support for mitigation measures and trust, including in governments, science, and pharmaceutical companies (4–6), suggests that differences in support between population subgroups may reflect differences in social trust between these groups.

Several limitations of the study need to be noted. Although our study comprised a large number of participants, the sample was not representative of the population of The Netherlands. Women, middle-aged people, higher educated people, people living with a family, people living in urban areas and people born in the Netherlands were overrepresented. Recruitment and data collection were conducted online. While 98% of households in the Netherlands have access to the internet at home (23), differences in digital literacy may have affected participation. Self-selection may have resulted in an overrepresentation of people who support COVID-19 mitigation measures, which may have been compounded by the periodic recruitment of new participants into the open cohort. The use of self-report to assess support for COVID-19 may have resulted in social desirability bias, albeit that data collection was anonymous. We only assessed support for the, arguably critical, mitigation measures that rely on individuals' voluntary behavior change. Support for institutional public health measures that directly limit people's options (e.g., closure of so-called non-essential shops) may differ substantially (2). In addition, we monitored support for mitigation measures only over the first year of the pandemic and a longer period of follow-up may be needed to identify pandemic fatigue.

## Conclusions

Despite suggestions of people growing increasingly tired of COVID-19-related restrictions (19–21), we found that public support in the first year of the pandemic response in the Netherlands decreased only for some COVID-19 mitigation measures and this decrease was more likely in some population subgroups than others, mostly in younger people. Our findings highlight that pandemic fatigue is not the presumed inevitable and natural response to a prolonged public health crisis (18), as has also been noted by others (19). Our findings, rather, point to the importance of understanding and addressing the factors that may influence support for specific mitigation measures, including the perceived balance of the expected benefits of COVID-19 mitigation measures and their potential cost, as well as social trust. As the perceived threat of the COVID-19 pandemic evolves over time, perceived trade-offs change as well, and support for measures will vary as a result, as they will in response to changes in social trust. It is critical that governments and public health and protection authorities clearly communicate the need for specific mitigation measures, ensure transparency and fairness of decisions, and put policies and services in place to counteract any negative impacts (e.g., mentally, socially, and financially) that mitigation measures may have, especially for specific groups.

## Data availability statement

The data analyzed in this study is subject to the following licenses/restrictions: the datasets used and/or analyzed during the current study are available from the corresponding author on reasonable request. Requests to access these datasets should be directed to j.dewit@uu.nl.

## Ethics statement

The study protocol was evaluated by the Centre for Clinical Expertise of the National Institute for Public Health and the Environment (RIVM) of the Netherlands (study number G&M-516). The study protocol was considered exempt from medical ethics review as governed by the Law on Research Involving Human Subjects (WMO) in The Netherlands and approved for implementation. The study was conducted in accordance with the Declaration of Helsinki of the World Medical Association and adhered to the General Data Protection Regulation and relevant codes of conduct of participating institutions. All participants provided informed consent for participation prior to completion of their first survey. After completing the first survey they provided informed consent for participation in the cohort study.

## Author contributions

JD, DR, BP, ML, and MB conceptualized the study. JD and DR wrote the original draft. ML and MB developed the study methodology and oversaw data collection. WB took carriage of data curation, with the support of FK and FS. WB analyzed the data, validated by FK and supervised by MB. ML and MB obtained the funding for the study. All authors contributed to the writing, reviewing and editing of the manuscript, and approved the submitted version. All authors agreed to be personally accountable for the author's own contributions and to ensure that questions related to the accuracy or integrity of any part of the work, including ones in which the author was not personally involved, are appropriately investigated, resolved, and the resolution documented in the literature.
