# Supplementary material for: Understanding public support for COVID-19 pandemic mitigation measures over time: Does it wear out?
Source: Front Public Health. 2023 Mar 3;11:1079992. doi: 10.3389/fpubh.2023.1079992 (PMC10020646; doi:10.3389/fpubh.2023.1079992)
Supplement: Supplementary file 1 [file Data_Sheet_1.docx]

**Understanding public support for COVID-19 pandemic mitigation measures over time: Does it wear out?**

**Supplementary Materials**

| **Supplementary Table 1. Participant enrollment and assessment of support for COVID-19 measures across waves** | | | | |
| --- | --- | --- | --- | --- |
| **Wave** | **Enrollment period** | **Number enrolled** | **Number in cohort** | **Number completing support items (N)** |
| 1 | 17 – 24 April 2020 | 89.943 |  | 29.157 |
| 2 | 7 – 12 May 2020 |  | 52.950 | 17.037 |
| 3 | 26 May – 1 June 2020 | 16.523 | 47.475 | 20.760 |
| 4 | 17 – 21 June 2020 |  | 50.290 | 16.110 |
| 5 | 8 – 12 July 2020 | 5.483 | 44.982 | 16.102 |
| 6 | 19 – 23 August 2020 | 17.185 | 44.466 | 18.951 |
| 7 | 30 September – 4 October 2020 |  | 47.740 | 15.186 |
| 8 | 11 – 15 November 2020 | 19.625 | 44.546 | 20.335 |
| 9 | 30 December 2020 – 3 January 2021 |  | 51.468 | 16.409 |
| 10 | 10 – 14 February 2021 | 8.622 | 45.741 | 17.503 |
| 11 | 24 – 28 March 2021 |  | 47.254 | 15.185 |
| 12 | 5 – 9 May 2021 | 11.283 | 44.159 | 17.773 |

| **Supplementary Table 2. Multivariable analysis assessing differences in trends in support for COVID-19 mitigation measures by participant characteristics** | | | | | | | | | | | | |
| --- | --- | --- | --- | --- | --- | --- | --- | --- | --- | --- | --- | --- |
|  |  | **Wave 1** | **Wave 2** | **Wave 3** | **Wave 4** | **Wave 6** | **Wave 7** | **Wave 8** | **Wave 9** | **Wave 10** | **Wave 11** | **Wave 12** |
| **Personal Hygiene** |  |  |  |  |  |  |  |  |  |  |  |  |
| Sex*Wave | Female | Reference | Reference | Reference | Reference | Reference | Reference | Reference | Reference | Reference | Reference | Reference |
|  | Male | Reference | .07 (.05-.08) | .05 (.03-.06) | .06 (.05-.08) | .06 (.04-.07) | .07 (.05-.08) | .06 (.04-.08) | .06 (.04-.08) | .05 (.04-.07) | .04 (.02-.05) | .03 (.01-.05) |
| Age*Wave | 70+ | Reference | Reference | Reference | Reference | Reference | Reference | Reference | Reference | Reference | Reference | Reference |
|  | 55-69y | Reference | -.04 (-.06--.02) | -.07 (-.09--.05) | -.05 (-.07--.03) | -.04 (-.06--.02) | -.06 (-.08--.04) | -.04 (-.06--.01) | -.07 (-.10--.05) | -.06 (-.09--.03) | -.08 (-.10--.05) | -.08 (-.11--.05) |
|  | 40-54y | Reference | -.07 (-.09--.05) | -.12 (-.14--.10) | -.07 (-.09--.05) | -.06 (-.09--.04) | -.09 (-.12--.07) | -.08 (-.10--.06) | -.11 (-.13--.08) | -.10 (-.13--.08) | -.10 (-.13--.08) | -.16 (-.19--.13) |
|  | 25-39y | Reference | -.07 (-.10--.05) | -.12 (-.14--.09) | -.04 (-.06--.01) | -.04 (-.06--.01) | -.07 (-.10--.05) | -.07 (-.10--.04) | -.09 (-.12--.06) | -.06 (-.09--.03) | -.09 (-.12--.06) | -.14 (-.17--.11) |
|  | 16-24y | Reference | -.05 (-.11-.01) | -.11 (-.16--.05) | -.04 (-.11-.03) | .01 (-.05-.07) | -.03 (-.09-.04) | -.06 (-.12--.01) | -.09 (-.16--.03) | -.003 (-.07-.06) | -.04 (-.11-.03) | -.06 (-.13-.01) |
| Educational level*Wave | Low | Reference | Reference | Reference | Reference | Reference | Reference | Reference | Reference | Reference | Reference | Reference |
|  | Middle | Reference | -.02 (-.04-.01) | -.02 (-.05-.003) | -.01 (-.04-.02) | -.02 (-.04-.01) | -.02 (-.05-.005) | -.01 (-.04-.02) | -.01 (-.04-.02) | -.03 (-.06-.01) | -.01 (-.04-.03) | -.05 (-.08--.02) |
|  | High | Reference | .01 (-.01-.03) | .04 (.01-.06) | .05 (.03-.07) | .02 (-.01-.04) | .01 (-.02-.04) | .02 (-.003-.05) | .02 (-.01-.04) | .02 (-.01-.05) | .03 (.001-.06) | -.01 (-.04-.02) |
| Country of birth*Wave | The Netherlands | Reference | Reference | Reference | Reference | Reference | Reference | Reference | Reference | Reference | Reference | Reference |
|  | Other | Reference | -.002 (-.03-.03) | -.01 (-.04-.03) | -.003 (-.04-.03) | .01 (-.03-.05) | .01 (-.03-.05) | .02 (-.02-.05) | .02 (-.02-.06) | .03 (-.01-.07) | .06 (.01-.10) | .06 (.01-.10) |
| Living alone*Wave | No | Reference | Reference | Reference | Reference | Reference | Reference | Reference | Reference | Reference | Reference | Reference |
|  | Yes | Reference | .01 (-.01-.03) | .01 (-.01-.03) | -.01 (-.02-.01) | -.01 (-.03-.01) | -.0003 (-.02-.02) | .003 (-.02-.02) | -.001 (-.02-.02) | -.0003 (-.02-.02) | -.01 (-.03-.01) | -.004 (-.03-.02) |
| Medical condition*Wave | No | Reference | Reference | Reference | Reference | Reference | Reference | Reference | Reference | Reference | Reference | Reference |
|  | Yes | Reference | .02 (.003-.04) | .03 (.01-.04) | .01 (-.01-.03) | .02 (-0.0000-.04) | .02 (-.003-.03) | .01 (-.005-.03) | .01 (-.01-.03) | .02 (.001-.04) | -.001 (-.02-.02) | .04 (.02-.06) |
| **Not Shaking Hands** |  |  |  |  |  |  |  |  |  |  |  |  |
| Sex*Wave | Female | Reference | Reference | Reference | Reference | Reference | Reference | Reference | Reference | Reference | Reference | Reference |
|  | Male | Reference | .07 (.05-.10) | .07 (.04-.10) | .06 (.03-.09) | .07 (.04-.10) | .11 (.08-.14) | .10 (.07-.13) | .14 (.11-.18) | .12 (.09-.15) | .12 (.08-.15) | .13 (.10-.17) |
| Age*Wave | 70+ | Reference | Reference | Reference | Reference | Reference | Reference | Reference | Reference | Reference | Reference | Reference |
|  | 55-69y | Reference | -.11 (-.15--.07) | -.13 (-.17--.09) | -.10 (-.14--.06) | -.13 (-.17--.09) | -.12 (-.16--.08) | -.17 (-.22--.13) | -.23 (-.27--.18) | -.20 (-.25--.16) | -.24 (-.28--.19) | -.32 (-.36--.27) |
|  | 40-54y | Reference | -.16 (-.20--.12) | -.26 (-.30--.22) | -.23 (-.27--.19) | -.25 (-.29--.21) | -.24 (-.29--.20) | -.30 (-.34--.26) | -.40 (-.44--.35) | -.37 (-.41--.32) | -.40 (-.45--.35) | -.60 (-.65--.55) |
|  | 25-39y | Reference | -.20 (-.25--.15) | -.32 (-.37--.27) | -.27 (-.32--.22) | -.34 (-.39--.29) | -.36 (-.41--.31) | -.42 (-.47--.37) | -.50 (-.55--.45) | -.47 (-.52--.41) | -.54 (-.60--.49) | -.72 (-.77--.66) |
|  | 16-24y | Reference | -.22 (-.33--.11) | -.27 (-.37--.16) | -.24 (-.37--.11) | -.37 (-.47--.27) | -.34 (-.46--.22) | -.42 (-.52--.33) | -.50 (-.61--.38) | -.40 (-.52--.29) | -.51 (-.63--.39) | -.73 (-.85--.60) |
| Educational level*Wave | Low | Reference | Reference | Reference | Reference | Reference | Reference | Reference | Reference | Reference | Reference | Reference |
|  | Middle | Reference | -.05 (-.10--.005) | -.12 (-.17--.07) | -.06 (-.11--.01) | -.12 (-.17--.07) | -.11 (-.16--.06) | -.15 (-.20--.10) | -.16 (-.22--.11) | -.22 (-.27--.16) | -.22 (-.27--.16) | -.31 (-.37--.25) |
|  | High | Reference | -.09 (-.14--.05) | -.10 (-.14--.06) | -.07 (-.11--.02) | -.14 (-.18--.09) | -.16 (-.21--.11) | -.16 (-.21--.11) | -.22 (-.27--.17) | -.23 (-.28--.18) | -.25 (-.30--.20) | -.32 (-.38--.27) |
| Country of birth*Wave | The Netherlands | Reference | Reference | Reference | Reference | Reference | Reference | Reference | Reference | Reference | Reference | Reference |
|  | Other | Reference | -.03 (-.09-.03) | -.07 (-.13--.01) | .01 (-.05-.08) | -.02 (-.08-.05) | -.06 (-.13-.01) | -.01 (-.08-.06) | -.05 (-.12-.02) | -.02 (-.09-.06) | -.001 (-.08-.08) | .004 (-.07-.08) |
| Living alone*Wave | No | Reference | Reference | Reference | Reference | Reference | Reference | Reference | Reference | Reference | Reference | Reference |
|  | Yes | Reference | -.03 (-.06-.01) | -.01 (-.04-.03) | -.03 (-.07-.002) | -.02 (-.05-.02) | -.02 (-.06-.02) | -.03 (-.07-.01) | -.01 (-.05-.03) | -.0002 (-.04-.04) | .0003 (-.04-.04) | .01 (-.03-.06) |
| Medical condition*Wave | No | Reference | Reference | Reference | Reference | Reference | Reference | Reference | Reference | Reference | Reference | Reference |
|  | Yes | Reference | .05 (.02-.08) | .07 (.04-.10) | .03 (-.004-.06) | .07 (.03-.10) | .06 (.02-.09) | .09 (.05-.12) | .09 (.05-.12) | .07 (.03-.10) | .10 (.06-.13) | .13 (.09-.16) |
| **Testing & Self-Isolation** |  |  |  |  |  |  |  |  |  |  |  |  |
| Sex*Wave | Female |  |  |  | Reference | Reference | Reference | Reference | Reference | Reference | Reference | Reference |
|  | Male |  |  |  | Reference | -.01 (-.03-.01) | -.02 (-.04--.0003) | -.06 (-.08--.04) | -.09 (-.11--.07) | -.08 (-.10--.06) | -.07 (-.09--.04) | -.08 (-.11--.05) |
| Age*Wave | 70+ |  |  |  | Reference | Reference | Reference | Reference | Reference | Reference | Reference | Reference |
|  | 55-69y |  |  |  | Reference | -.01 (-.04-.02) | -.01 (-.04-.02) | -.02 (-.05-.01) | -.0004 (-.03-.03) | .03 (-.01-.06) | .02 (-.01-.05) | -.03 (-.07-.002) |
|  | 40-54y |  |  |  | Reference | -.02 (-.05-.01) | -.04 (-.07--.01) | .01 (-.03-.04) | .05 (.01-.08) | .06 (.02-.09) | .04 (.01-.08) | -.02 (-.06-.01) |
|  | 25-39y |  |  |  | Reference | .01 (-.02-.05) | -.004 (-.04-.03) | .02 (-.02-.06) | .11 (.07-.14) | .10 (.06-.14) | .02 (-.02-.06) | -.02 (-.06-.02) |
|  | 16-24y |  |  |  | Reference | -.09 (-.18-.01) | -.05 (-.16-.05) | -.04 (-.13-.06) | .03 (-.08-.13) | .05 (-.05-.15) | .03 (-.08-.14) | .02 (-.09-.13) |
| Educational level*Wave | Low |  |  |  | Reference | Reference | Reference | Reference | Reference | Reference | Reference | Reference |
|  | Middle |  |  |  | Reference | .01 (-.03-.04) | -.01 (-.04-.03) | -.01 (-.05-.03) | .01 (-.03-.05) | -.01 (-.05-.03) | .001 (-.04-.04) | -.04 (-.09-.001) |
|  | High |  |  |  | Reference | .04 (.01-.08) | .01 (-.03-.04) | .02 (-.02-.05) | .02 (-.01-.06) | .04 (.01-.08) | .04 (.001-.08) | .02 (-.02-.06) |
| Country of birth*Wave | The Netherlands |  |  |  | Reference | Reference | Reference | Reference | Reference | Reference | Reference | Reference |
|  | Other |  |  |  | Reference | -.03 (-.08-.02) | -.01 (-.06-.04) | -.05 (-.10-.01) | -.07 (-.13--.02) | -.03 (-.09-.02) | .01 (-.05-.06) | .02 (-.04-.07) |
| Living alone*Wave | No |  |  |  | Reference | Reference | Reference | Reference | Reference | Reference | Reference | Reference |
|  | Yes |  |  |  | Reference | -.01 (-.04-.01) | -.02 (-.05-.01) | -.02 (-.05-.004) | -.02 (-.05-.01) | -.02 (-.05-.01) | -.001 (-.03-.03) | .01 (-.02-.04) |
| Medical condition*Wave | No |  |  |  | Reference | Reference | Reference | Reference | Reference | Reference | Reference | Reference |
|  | Yes |  |  |  | Reference | .003 (-.02-.03) | .02 (-.003-.05) | .01 (-.02-.03) | -.01 (-.04-.01) | -.01 (-.03-.02) | .004 (-.02-.03) | .01 (-.02-.03) |
| **Quarantine** |  |  |  |  |  |  |  |  |  |  |  |  |
| Sex*Wave | Female |  |  |  |  | Reference | Reference | Reference | Reference | Reference | Reference | Reference |
|  | Male |  |  |  |  | Reference | -.05 (-.07--.03) | -.03 (-.05--.01) | -.03 (-.05--.01) | -.05 (-.07--.03) | -.04 (-.07--.02) | -.04 (-.07--.02) |
| Age*Wave | 70+ |  |  |  |  | Reference | Reference | Reference | Reference | Reference | Reference | Reference |
|  | 55-69y |  |  |  |  | Reference | -.003 (-.03-.03) | -.01 (-.05-.02) | .01 (-.02-.04) | .05 (.02-.09) | .02 (-.02-.05) | -.02 (-.06-.01) |
|  | 40-54y |  |  |  |  | Reference | .05 (.02-.08) | .02 (-.01-.05) | .03 (-.01-.06) | .10 (.06-.13) | .05 (.01-.08) | -.06 (-.10--.02) |
|  | 25-39y |  |  |  |  | Reference | .09 (.06-.13) | .002 (-.03-.04) | .04 (.002-.07) | .10 (.07-.14) | .03 (-.01-.07) | -.09 (-.13--.05) |
|  | 16-24y |  |  |  |  | Reference | .12 (.04-.20) | -.03 (-.09-.04) | .02 (-.06-.10) | .12 (.04-.19) | .10 (.01-.18) | -.07 (-.16-.01) |
| Educational level*Wave | Low |  |  |  |  | Reference | Reference | Reference | Reference | Reference | Reference | Reference |
|  | Middle |  |  |  |  | Reference | .04 (.002-.08) | -.02 (-.06-.02) | .03 (-.01-.07) | .05 (.005-.09) | .04 (-.004-.08) | -.01 (-.05-.04) |
|  | High |  |  |  |  | Reference | .04 (.001-.07) | .01 (-.02-.05) | .08 (.04-.12) | .13 (.10-.17) | .10 (.06-.14) | .08 (.04-.13) |
| Country of birth*Wave | The Netherlands |  |  |  |  | Reference | Reference | Reference | Reference | Reference | Reference | Reference |
|  | Other |  |  |  |  | Reference | .07 (.02-.13) | .004 (-.05-.05) | -.01 (-.07-.04) | .04 (-.01-.09) | .04 (-.01-.10) | -.01 (-.07-.05) |
| Living alone*Wave | No |  |  |  |  | Reference | Reference | Reference | Reference | Reference | Reference | Reference |
|  | Yes |  |  |  |  | Reference | .003 (-.02-.03) | -.02 (-.04-.01) | .002 (-.03-.03) | .02 (-.01-.05) | .0003 (-.03-.03) | .02 (-.02-.05) |
| Medical condition*Wave | No |  |  |  |  | Reference | Reference | Reference | Reference | Reference | Reference | Reference |
|  | Yes |  |  |  |  | Reference | -.01 (-.04-.01) | -.03 (-.05--.01) | -.07 (-.09--.04) | -.07 (-.09--.04) | -.08 (-.11--.05) | -.03 (-.05-.003) |
| **Restricting Social Contacts** |  |  |  |  |  |  |  |  |  |  |  |  |
| Sex*Wave | Female |  | Reference | Reference | Reference | Reference | Reference | Reference | Reference | Reference | Reference | Reference |
|  | Male |  | Reference | .03 (.01-.05) | .07 (.05-.09) | .05 (.03-.07) | .03 (.01-.05) | .05 (.02-.07) | .04 (.02-.07) | .06 (.03-.08) | .09 (.07-.12) | .08 (.05-.11) |
| Age*Wave | 70+ |  | Reference | Reference | Reference | Reference | Reference | Reference | Reference | Reference | Reference | Reference |
|  | 55-69y |  | Reference | -.04 (-.07--.01) | -.08 (-.11--.05) | -.02 (-.06-.01) | .01 (-.02-.04) | -.03 (-.06-.004) | -.01 (-.04-.02) | .02 (-.02-.05) | -.02 (-.06-.02) | -.08 (-.12--.05) |
|  | 40-54y |  | Reference | -.13 (-.16--.10) | -.18 (-.21--.15) | -.13 (-.16--.10) | -.05 (-.09--.02) | -.10 (-.13--.06) | -.09 (-.12--.05) | -.07 (-.10--.03) | -.12 (-.16--.09) | -.27 (-.30--.23) |
|  | 25-39y |  | Reference | -.13 (-.16--.10) | -.21 (-.24--.17) | -.19 (-.23--.15) | -.13 (-.17--.10) | -.18 (-.21--.14) | -.14 (-.18--.11) | -.15 (-.19--.11) | -.28 (-.33--.24) | -.39 (-.43--.34) |
|  | 16-24y |  | Reference | -.15 (-.23--.07) | -.30 (-.39--.21) | -.31 (-.39--.22) | -.27 (-.37--.18) | -.28 (-.37--.19) | -.26 (-.35--.16) | -.22 (-.31--.12) | -.41 (-.51--.31) | -.50 (-.61--.40) |
| Educational level*Wave | Low |  | Reference | Reference | Reference | Reference | Reference | Reference | Reference | Reference | Reference | Reference |
|  | Middle |  | Reference | -.02 (-.06-.01) | -.04 (-.08--.01) | -.05 (-.09--.01) | -.01 (-.05-.03) | -.03 (-.07-.01) | -.03 (-.07-.02) | -.03 (-.07-.01) | -.04 (-.09-.0003) | -.11 (-.15--.06) |
|  | High |  | Reference | .04 (.004-.07) | -.01 (-.04-.02) | -.04 (-.07--.001) | -.02 (-.06-.02) | -.02 (-.05-.02) | -.03 (-.07-.01) | -.004 (-.04-.03) | -.02 (-.07-.02) | -.07 (-.11--.03) |
| Country of birth*Wave | The Netherlands |  | Reference | Reference | Reference | Reference | Reference | Reference | Reference | Reference | Reference | Reference |
|  | Other |  | Reference | .04 (.0001-.09) | .07 (.02-.11) | .03 (-.02-.07) | .01 (-.04-.06) | -.01 (-.06-.04) | .01 (-.04-.06) | .03 (-.02-.08) | .04 (-.01-.10) | .07 (.01-.12) |
| Living alone*Wave | No |  | Reference | Reference | Reference | Reference | Reference | Reference | Reference | Reference | Reference | Reference |
|  | Yes |  | Reference | .03 (.001-.05) | .04 (.01-.06) | .005 (-.02-.03) | -.01 (-.04-.02) | .002 (-.03-.03) | .02 (-.01-.04) | .03 (-.004-.05) | .03 (-.01-.06) | .04 (.01-.07) |
| Medical condition*Wave | No |  | Reference | Reference | Reference | Reference | Reference | Reference | Reference | Reference | Reference | Reference |
|  | Yes |  | Reference | .02 (-.01-.04) | .04 (.02-.07) | .02 (-.002-.05) | -.01 (-.03-.02) | -.01 (-.03-.02) | -.01 (-.04-.01) | .003 (-.02-.03) | .01 (-.02-.04) | .05 (.02-.07) |
| **Limiting Visitors at Home** |  |  |  |  |  |  |  |  |  |  |  |  |
| Sex*Wave | Female | Reference | Reference | Reference |  | Reference | Reference | Reference | Reference | Reference | Reference | Reference |
|  | Male | Reference | .10 (.07-.13) | .14 (.11-.17) |  | .17 (.14-.20) | .17 (.14-.20) | .24 (.20-.27) | .16 (.13-.20) | .29 (.25-.32) | .35 (.31-.39) | .23 (.19-.27) |
| Age*Wave | 70+ | Reference | Reference | Reference |  | Reference | Reference | Reference | Reference | Reference | Reference | Reference |
|  | 55-69y | Reference | -.16 (-.20--.11) | -.21 (-.25--.16) |  | -.25 (-.30--.20) | -.24 (-.28--.19) | -.32 (-.37--.28) | -.27 (-.32--.22) | -.39 (-.44--.34) | -.41 (-.46--.35) | -.39 (-.44--.34) |
|  | 40-54y | Reference | -.25 (-.29--.21) | -.39 (-.43--.35) |  | -.54 (-.59--.50) | -.46 (-.51--.41) | -.63 (-.67--.58) | -.55 (-.60--.50) | -.69 (-.74--.64) | -.72 (-.77--.66) | -.75 (-.80--.70) |
|  | 25-39y | Reference | -.32 (-.37--.27) | -.50 (-.55--.45) |  | -.75 (-.80--.70) | -.69 (-.75--.64) | -.95 (-1.01--.90) | -.76 (-.82--.71) | -1.10 (-1.16--1.04) | -1.20 (-1.26--1.14) | -1.04 (-1.10--.98) |
|  | 16-24y | Reference | -.26 (-.39--.14) | -.48 (-.59--.37) |  | -.94 (-1.05--.83) | -.87 (-1.00--.73) | -1.04 (-1.14--.94) | -.86 (-.99--.74) | -1.01 (-1.13--.89) | -1.04 (-1.18--.91) | -1.14 (-1.28--1.00) |
| Educational level*Wave | Low | Reference | Reference | Reference |  | Reference | Reference | Reference | Reference | Reference | Reference | Reference |
|  | Middle | Reference | -.15 (-.20--.10) | -.19 (-.24--.14) |  | -.27 (-.33--.22) | -.24 (-.30--.18) | -.38 (-.44--.32) | -.31 (-.37--.24) | -.38 (-.44--.31) | -.39 (-.46--.33) | -.35 (-.42--.28) |
|  | High | Reference | -.17 (-.22--.12) | -.19 (-.24--.15) |  | -.24 (-.29--.19) | -.24 (-.30--.19) | -.38 (-.43--.33) | -.29 (-.35--.23) | -.42 (-.48--.36) | -.46 (-.52--.40) | -.36 (-.42--.30) |
| Country of birth*Wave | The Netherlands | Reference | Reference | Reference |  | Reference | Reference | Reference | Reference | Reference | Reference | Reference |
|  | Other | Reference | .02 (-.05-.08) | .15 (.08-.21) |  | .03 (-.04-.10) | .10 (.02-.17) | .10 (.02-.17) | .02 (-.06-.10) | .13 (.05-.21) | .17 (.08-.25) | .12 (.03-.20) |
| Living alone*Wave | No | Reference | Reference | Reference |  | Reference | Reference | Reference | Reference | Reference | Reference | Reference |
|  | Yes | Reference | .11 (.07-.14) | .19 (.15-.23) |  | .16 (.12-.20) | .19 (.15-.23) | .18 (.14-.22) | .15 (.11-.20) | .30 (.25-.34) | .35 (.30-.40) | .20 (.15-.25) |
| Medical condition*Wave | No | Reference | Reference | Reference |  | Reference | Reference | Reference | Reference | Reference | Reference | Reference |
|  | Yes | Reference | .14 (.10-.17) | .17 (.13-.20) |  | .15 (.12-.19) | .12 (.08-.16) | .15 (.12-.19) | .10 (.06-.14) | .21 (.17-.25) | .20 (.16-.24) | .18 (.14-.22) |
| **Not Traveling Abroad** |  |  |  |  |  |  |  |  |  |  |  |  |
| Sex*Wave | Female | Reference | Reference | Reference |  |  |  | Reference | Reference | Reference | Reference | Reference |
|  | Male | Reference | .04 (.01-.07) | .05 (.02-.08) |  |  |  | .10 (.07-.14) | .13 (.09-.17) | .12 (.09-.16) | .16 (.12-.20) | .17 (.13-.21) |
| Age*Wave | 70+ | Reference | Reference | Reference |  |  |  | Reference | Reference | Reference | Reference | Reference |
|  | 55-69y | Reference | -.19 (-.24--.15) | -.25 (-.30--.21) |  |  |  | -.26 (-.30--.21) | -.27 (-.32--.22) | -.25 (-.30--.20) | -.34 (-.39--.29) | -.41 (-.47--.36) |
|  | 40-54y | Reference | -.26 (-.31--.22) | -.44 (-.48--.39) |  |  |  | -.47 (-.52--.42) | -.52 (-.57--.47) | -.49 (-.54--.44) | -.63 (-.69--.58) | -.78 (-.83--.73) |
|  | 25-39y | Reference | -.33 (-.37--.28) | -.46 (-.51--.41) |  |  |  | -.65 (-.71--.60) | -.65 (-.71--.59) | -.64 (-.70--.58) | -.84 (-.90--.78) | -1.04 (-1.11--.98) |
|  | 16-24y | Reference | -.28 (-.40--.16) | -.43 (-.55--.32) |  |  |  | -.68 (-.78--.57) | -.68 (-.80--.55) | -.66 (-.79--.54) | -.89 (-1.02--.76) | -1.08 (-1.21--.94) |
| Educational level*Wave | Low | Reference | Reference | Reference |  |  |  | Reference | Reference | Reference | Reference | Reference |
|  | Middle | Reference | -.13 (-.18--.08) | -.19 (-.24--.14) |  |  |  | -.25 (-.31--.20) | -.27 (-.33--.21) | -.32 (-.38--.26) | -.38 (-.44--.31) | -.41 (-.48--.35) |
|  | High | Reference | -.20 (-.25--.16) | -.26 (-.30--.21) |  |  |  | -.36 (-.42--.31) | -.40 (-.46--.35) | -.40 (-.46--.35) | -.51 (-.57--.45) | -.54 (-.60--.49) |
| Country of birth*Wave | The Netherlands | Reference | Reference | Reference |  |  |  | Reference | Reference | Reference | Reference | Reference |
|  | Other | Reference | -.10 (-.17--.03) | .002 (-.06-.07) |  |  |  | -.23 (-.30--.15) | -.20 (-.28--.12) | -.20 (-.28--.12) | -.19 (-.27--.10) | -.23 (-.32--.15) |
| Living alone*Wave | No | Reference | Reference | Reference |  |  |  | Reference | Reference | Reference | Reference | Reference |
|  | Yes | Reference | .04 (-.001-.08) | .09 (.05-.13) |  |  |  | .05 (.01-.09) | .07 (.03-.11) | .06 (.02-.11) | .12 (.07-.17) | .15 (.11-.20) |
| Medical condition*Wave | No | Reference | Reference | Reference |  |  |  | Reference | Reference | Reference | Reference | Reference |
|  | Yes | Reference | .10 (.06-.13) | .16 (.12-.19) |  |  |  | .18 (.15-.22) | .15 (.11-.18) | .17 (.13-.20) | .19 (.15-.24) | .25 (.20-.29) |
| **Wearing Face Mask** |  |  |  |  |  |  |  |  |  |  |  |  |
| Sex*Wave | Female |  |  |  |  |  | Reference | Reference | Reference | Reference | Reference | Reference |
|  | Male |  |  |  |  |  | Reference | .05 (.02-.09) | .07 (.04-.10) | .06 (.03-.10) | .07 (.04-.11) | .08 (.05-.12) |
| Age*Wave | 70+ |  |  |  |  |  | Reference | Reference | Reference | Reference | Reference | Reference |
|  | 55-69y |  |  |  |  |  | Reference | .02 (-.02-.06) | -.01 (-.05-.03) | .05 (.01-.10) | .03 (-.02-.07) | -.03 (-.07-.02) |
|  | 40-54y |  |  |  |  |  | Reference | .07 (.02-.11) | .005 (-.04-.05) | .11 (.07-.16) | .08 (.03-.13) | -.07 (-.12--.02) |
|  | 25-39y |  |  |  |  |  | Reference | .04 (-.01-.09) | -.005 (-.06-.05) | .09 (.04-.15) | .06 (.003-.12) | -.10 (-.16--.04) |
|  | 16-24y |  |  |  |  |  | Reference | .06 (-.07-.19) | .03 (-.11-.16) | .10 (-.03-.24) | .10 (-.05-.24) | -.11 (-.25-.04) |
| Educational level*Wave | Low |  |  |  |  |  | Reference | Reference | Reference | Reference | Reference | Reference |
|  | Middle |  |  |  |  |  | Reference | -.04 (-.09-.02) | -.04 (-.09-.02) | -.002 (-.06-.06) | .001 (-.06-.06) | -.04 (-.10-.03) |
|  | High |  |  |  |  |  | Reference | .02 (-.03-.07) | .01 (-.04-.06) | .10 (.05-.16) | .10 (.05-.15) | .07 (.01-.12) |
| Country of birth*Wave | The Netherlands |  |  |  |  |  | Reference | Reference | Reference | Reference | Reference | Reference |
|  | Other |  |  |  |  |  | Reference | -.04 (-.11-.04) | -.01 (-.08-.07) | .01 (-.07-.08) | .002 (-.07-.08) | -.01 (-.09-.07) |
| Living alone*Wave | No |  |  |  |  |  | Reference | Reference | Reference | Reference | Reference | Reference |
|  | Yes |  |  |  |  |  | Reference | -.003 (-.04-.04) | .02 (-.02-.06) | .01 (-.03-.05) | .01 (-.03-.05) | .03 (-.01-.07) |
| Medical condition*Wave | No |  |  |  |  |  | Reference | Reference | Reference | Reference | Reference | Reference |
|  | Yes |  |  |  |  |  | Reference | -.04 (-.08--.01) | -.05 (-.09--.02) | -.08 (-.12--.04) | -.08 (-.11--.04) | -.04 (-.08--.0004) |
